# A green, fast protocol to estimate the accumulation of airborne anthropogenic microfibers in *Pittosporum tobira* in urban areas: effects of season and rainfall

**DOI:** 10.7717/peerj.20558

**Published:** 2026-01-14

**Authors:** Anna Gaglione, Angelo Granata, Fiore Capozzi, Antonio Rallo, Simonetta Giordano, Maria Cristina Sorrentino, Valeria Spagnuolo

**Affiliations:** Department of Biology, University of Naples Federico II, Naples, Italy

**Keywords:** Microplastics, Bioaccumulation, Environmental pollution, Organic polymers, Evergreen plant, Biomonitors

## Abstract

Plastics represent a major organic pollutant, but research focused on their biomonitoring in the air has only recently received attention. In the present work, we investigated the ability of *Pittosporum tobira* leaves to distinguish different levels of air contamination due to anthropogenic microfibers (MFs) in six urban sites characterized by different land uses (industrial, urban, and green), and the effect of wet/dry season on their accumulation. Moreover, the effect of pouring rain on MFs accumulation was estimated by transplants of *P. tobira*. Microfiber extraction was done by tape tearing on 1 g composite leaf samples on the leaf surface. In summer, the highest number of MFs were found in the leaves from the industrial site (160), followed by urban ones (84–125), and green parks (48–54). The accumulation of MFs was overall higher in summer than in winter, due to the rain-washing effect in the latter, and the different leaf traits observed in the two seasons. The development of glandular hairs during summer could contribute to increasing the accumulation of MFs observed in this period under conditions of reduced precipitation. In agreement, when comparing MFs fallout on leaves of sheltered and unsheltered transplants after a heavy rainfall, the number counted on the latter was significantly lower, suggesting that precipitations reduce MFs deposition. These findings reinforce the suitability of *Pittosporum tobira* leaves as a bioindicator for airborne anthropogenic MFs. Moreover, the pronounced seasonal differences, as well as the higher MFs loads during dry summer months, indicate that monitoring sensitivity is enhanced under low-rainfall conditions.

## Introduction

Plastic is considered one of the major environmental persistent pollutants (Adeniran, Ayesu-Koranteng & Shakantu, 2022; Williams & Rangel-Buitrago, 2022). Being elastic, durable, lightweight, waterproof, cheap and easy to manufacture, synthetic polymers have become the protagonists in several markets, as packaging, textiles, construction materials, and industrial equipment. In 2021, world plastic production was estimated at 390 million tons - of which 90% derived from fossil sources - and China, with one third of the global production, was the largest plastic manufacturer in the world. Since the demand for plastics is predictably growing, and its degradation requests from decades to millennia (Li, Tse & Fok, 2016; Mohan et al., 2022; Ward et al., 2019), the amount of plastic waste is destinated to increase as well (Rednikin et al., 2024). Once produced, plastics undergo ageing and degradation, and break down in smaller fragments, forming meso- micro and nanoplastics which are added to primary micro- and nanoplastics, produced as such with various uses, *e.g.*, chemical abrasives (Rednikin et al., 2024).

Another worrying aspect is that plastic products have been found in the most inhospitable places of our planet (Citterich, Giudice & Azzaro, 2023) indicating that plastic contamination is ubiquitous and affects all environmental matrices. Indeed, it is reported that microplastics (MPs) (1 µm to five mm) (Da Costa Filho et al., 2021) and nanoplastics (NPs) (<1 μm) (Jakubowicz, Enebro & Yarahmadi, 2021) are widely underestimated (Cesarini et al., 2023; Taurozzi et al., 2024). Airborne MPs have been low-considered and studied so far in comparison with MPs dispersed in the ocean, sediments, and soil. Fibers are the predominant morphotype in pristine areas (Roblin & Aherne, 2020; Capozzi et al., 2023), and urban environments (Dris et al., 2015), both indoors (Amato-Lourenço et al., 2022; Chen et al., 2022) and outdoors (Wright et al., 2020).

Road traffic emissions (estimated as 7 million kg year^−1^) (Evangeliou et al., 2020), sea spray (Harb, Pokhrel & Foroutan, 2023), waste incineration, building materials, and synthetic fabrics (Amato-Lourenço et al., 2022) are considered the sources of airborne MPs giving the highest contribution. Their transport, distribution, and deposition is strongly affected by meteorological conditions and human activities (Paramitadevi et al., 2023; Habibi et al., 2022; Pandey et al., 2022).

As for other airborne pollutants, MPs concentrations in urban areas were recently monitored by using passive samplers (Cai et al., 2017; Dris et al., 2016; Leonard et al., 2023; Zhou, Tian & Luo, 2017). However, these devices are expensive and require specific expertise to perform sampling properly. The role of plants as biomonitors of airborne pollutants is grounded on their high surface to mass ratio, and their ability of intercepting particulate matter and related pollutants (Di Palma et al., 2017; Capozzi et al., 2020a). Thus, plant leaves represent a good alternative to passive samplers, and could potentially be used as a biomonitoring system also for airborne anthropogenic microfibers (MFs), for which a simple and reproducible protocol is still missing.

To fill this gap, in previous work we investigated the ability of *Pittosporum tobira* leaves to intercept and retain airborne MFs and set up a 4-steps sequential extraction procedure (Capozzi et al., 2024). We found that the percentage of particles, mostly fibers, extracted at each step was constant in all samples. Moreover, the first step of the sequential procedure (consisting in a tape tearing on the leaf surface) extracted the largest fraction of MFs, about 75%. Therefore, in the present work we hypothesized that *P. tobira* leaves could be sensitive enough to distinguish different levels of pollution due to airborne MFs, even by tape tearing alone. Also, given that most MFs are found on the leaf surface, we hypothesized that their accumulation could be influenced by rainfall regime. To test these hypotheses, we carried out a biomonitoring survey along an urban pollution gradient, analyzing *Pittosporum tobira* leaves sampled in six urban sites during summer (dry) and winter (rainy season) to investigate possible seasonal variation in their accumulation. Leaf traits of samples collected during both summer and winter, were examined by scanning electron microscope (SEM), as well. Finally, to further investigate the effect of pouring rain, *P. tobira* transplants (*i.e.,* plants grown in pots), here used for the first time, were exposed sheltered, to protect them from the rain, and unsheltered, and their MFs accumulation was compared after heavy rain.

## Materials & Methods

### Plant material and study area

*Pittorposum tobira* (Thunb.) W.T.Aiton is a subtropical evergreen shrub, native to Japan, Korea, Nansei-shoto, and Vietnam, but introduced as ornamental plant in temperate regions of Europe, USA, China and north Africa (https://powo.science.kew.org/taxon/urn:lsid:ipni.org:names:77227718-1). Here, this species is largely used in urban gardens due to its adaptability to variable environmental conditions (temperature fluctuations, rainfall regime) and plays a positive role in stabilizing soil and improving its quality (Pan et al., 2024). Pittosporum tobira leaves were sampled in six sites (Table S1) in Naples municipality, within an area of about 12 Km in diameter. The selected sites, characterized by different land uses, are all embedded in urban context.

### Sampling of native plants and transplants

Two different samplings were carried out, the first in July 2024 (summer, dry season), the second in December–January- (winter, wet season). During winter, leaf sampling at the six selected sites was done 2 weeks after a heavy rain event. We collected fully developed and not damaged leaves at 100 cm ± 10 cm above ground from plants found at each site. From 10 g-samples (about 20 leaves collected all around each shrub or hedge), three independent biological replicas of 1 g were composed by cutting and mixing leaf pieces of about 1.5 cm^2^ (for details, see Capozzi et al., 2024).

Moreover, in the wet season, an exposure of transplants (*i.e.,* plants of *P. tobira* growing in pots) was carried out to estimate the effect of heavy rain on the accumulation of MFs. To this purpose, six transplants of *P. tobira* were placed on the roof of the Department of Biology of the University Federico II of Naples, three of which fully exposed to rainfall and other three sheltered from the rain, and the leaves were analyzed immediately after pouring rain. This experiment was repeated 2 times using different transplants each time, and variable exposure times (10- and 15-days exposure), depending on the occurrence of a heavy rain event (>30 mm m^−2^ in the last 24 h). During the exposure period daily weather parameters (Table S2), specifically rainfall and wind speed, were recorded by the weather station next to the Department (https://www.meteo.unina.it/). In both periods, transplants were exposed on a day without precipitation and the leaves collected after heavy rainfall (34.8 mm). The winds had a comparable speed and were mostly weak in both periods (Table S2). Before exposure, transplants were cleaned by a jet of running tap water and then double-distilled water and dried with cool air. Leaf samples from cleaned transplants were considered as blank and the presence of MFs on their surface was assessed, as well. Transplants were watered 2 times a week. For all leaf samples, both from native and transplanted plants, 1 g fresh leaf sample (*i.e.,* the leaf tissue weight of each replica) was composed by mixing pieces of 10 g leaves collected around the canopy at 1 m (±0.1 m) above ground.

### Extraction of MFs

To evaluate whether the sequential extraction procedure had good repeatability in both summer and winter, leaf samples from Urban_2 (Piazza degli Artisti) collected in summer and winter were analyzed in triplicate following the sequential extraction procedure reported in detail in Capozzi et al. (2024). In brief, 1 g composite leaf samples were subjected to tape tearing of upper and lower leaf surface, and the tape, fixed on baking paper, was examined under a stereomicroscope to count, and measure MFs. Then, the same composite leaf sample was subjected to three liquid extractions: by water, ethanol, and wet peroxide oxidation (Capozzi et al., 2024). At each extraction step, solutions were filtered under vacuum on glass filters, and these latter examined under a stereomicroscope for MFs counting and measuring. This preliminary test was set up to assess the repeatability of the extraction method in the two seasons and to confirm that tape tearing extracts the largest fraction of MFs, as already observed in the previous experiment carried out in summer (Capozzi et al., 2024). Once we had ascertained the repeatability of the method in both summer and winter, we decided to proceed only with tape tearing. Therefore, tape tearing was performed on leaves of native plants sampled at each site in summer and winter, as well as on leaves of transplants before and after cleaning, and before and after sheltered/unsheltered exposure. All samples were observed under a stereomicroscope (Leica Wild M8) equipped by a AmScope model MU 100–Digital Microscope Camera, for counting and measuring anthropogenic MFs, which were identified according to criteria defined by Windsor et al. (2019).

### Sample preparation for SEM

Small pieces (2–3 mm in length) of summer and winter leaves were fixed in glutaraldehyde 2.5%, dehydrated by graded alcohol concentrations and critical point drying. All chemicals used were provided by Sigma. Leaf pieces were mounted on aluminum stubs, gold coated and observed by a SEM-Energy Dispersive Spectrometry (EDS) model Jeol JSM5310. For counting epicuticular waxy structures observed on the winter leaves, 20 squares of 1 µm^2^ were randomly selected on the SEM images of the upper surface, 10 in areas highly enriched of these waxy structures, and other 10 in areas in which a reduced number of structures occurred (Fig. S1). For glandular hairs quantification, ten squares of 50 µm^2^ were randomly selected on the SEM images of the summer leaves. Average number of waxy structures and hairs and their standard deviation was calculated in Excel.

### Quality control

All the precautions already adopted in the previous work (Capozzi et al., 2024), were applied in this work to avoid MFs contamination during the experimental procedures. A quality control test was performed by examining under the stereomicroscope all materials (*i.e.,* pieces of tape and oven paper; glass filters after vacuum filtration of each solvent used in the procedure) to ensure that the contribution of MPs deposition during extraction and observations was negligible.

### Statistical analysis

The software IBM SPSS Statistics (Version 27.0, IBM Corp., Armonk, NY, USA, released 2020) and Excel for Windows were used for basic statistical calculations and comparisons.

*T*-test for independent samples was used to assess the statistical significance of differences in the number of MFs (dependent variable) based on the following independent variable: water washing for the transplant experiment.

A two-way analysis of variance (ANOVA) was performed to examine the main and interaction effects of land use type and season on the number of A-MFs. Multiple comparisons were performed using Tukey’s post hoc test (*p* < 0.05). The normality and homogeneity of variances were assessed using the Shapiro–Wilk test and Levene’s test, respectively. The nonparametric Kruskal-Wallis analysis of variance by ranks was used to compare the median number of MFs (dependent variables) in sheltered, unsheltered conditions (independent variables). In case of rejection of the null hypothesis, pairwise comparisons were performed using the Mann–Whitney U test (*p* < 0.05). The Mann–Whitney U test (*p* < 0.05) was also used to compare the median length of MFs (dependent variable) across different sites and seasons (independent variables).

## Results

### Quality control

The examination of all control samples, *i.e.,* the tape, the oven paper, the glass-fiber filters, the MilliQ water and all liquid reagents used during the extraction steps highlighted the presence of 1.73 ± 1.19 (mean and standard deviation (SD)) fibers over all samples; therefore, a negligible number of MFs could contaminate the samples during the extraction procedure.

### Sequential extraction procedure

The percentage of MFs collected at Urban_2 in summer and winter was constant at each season (Table 1). However, the proportion of MFs collected by tape tearing was higher in summer than in winter (77% *vs.* 68%); conversely, the fractions of MFs collected by liquid extraction steps, and particularly ethanol and wet peroxide oxidation, were higher in winter than in summer (8–9% *vs.* 5%).

**Table 1:** Mean ± SD (*n* = 3) and percentage on total number of MFs extracted by each step of the sequential approach (Capozzi et al., 2024).

**Winter**	**R1** ^*^	**R2**	**R3**	**Mean**	**SD**	**%**
T	70	65	72	69	3.6	68%
W	15	19	14	16	2.6	16%
E	8	10	8	9	1.2	9%
D	8	7	10	8	1.5	8%
**Summer**	**R1**	**R2**	**R3**	**Mean**	**SD**	**%**
T	120	133	125	126	6.6	77%
W	21	23	24	23	1.5	14%
E	6	8	9	8	1.5	5%
D	7	9	8	8	1.0	5%

**Notes.**

* R1, R2 and R3 are three leaf samples analysed by 4-step sequential extraction procedure; in each column the number of MFs counted on the total leaf surface (upper + lower) is reported.

T tape tearing W water extraction E ethanol extraction D wet peroxide oxidation

### Site comparison and seasonal variations

The two-way ANOVA analysis revealed statistically significant main effects for both independent variables, as well as a significant interaction:

• Main effect of land use type: significant main effect was found for land use type, F(5, 24) = 79.00, *p* < .001, indicating that the mean number of MFs differed significantly across the sites.
• Main effect of season: significant main effect was found for season, F(1, 24) = 241.00, *p* < .001. Overall, the mean microfiber count was higher during the summer than the winter season.
• Interaction effect: the two-way interaction between land use type and season was also statistically significant, F(5, 24) = 24.00, *p* < .001, indicating that the effect of the season on MF abundance is not uniform across all land use types.

**Figure 1:**
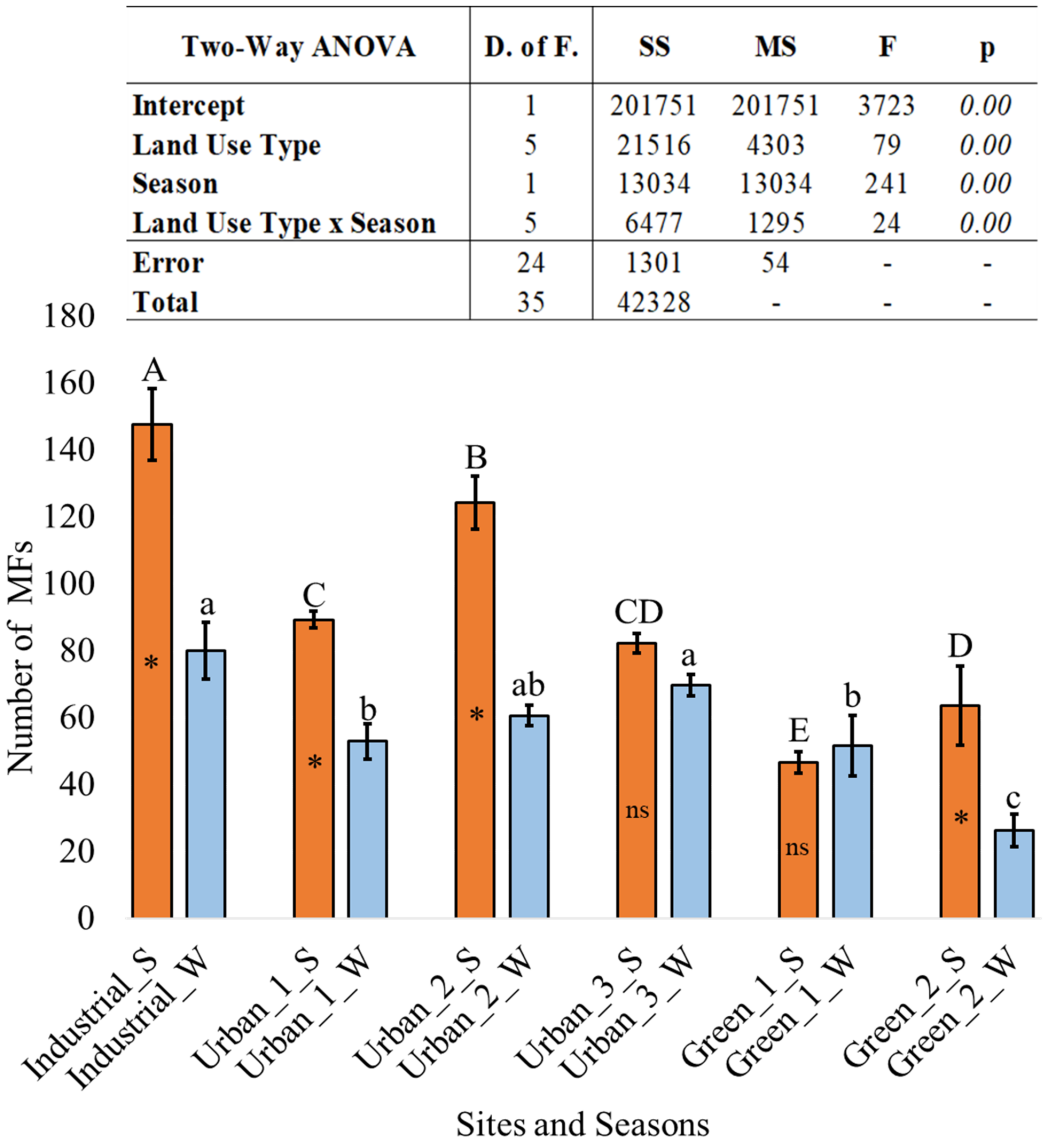
Two-way ANOVA output and bar chart of the number of MFs found at different sites and seasons. Summer “S” and winter “W”. Bars: means, error bars: standard deviations, *n* = 3. * = the higher value for “season effect”. Different letters indicate significant differences according to Tukey’s test (*p* < 0.05). ns: not significantly different.

To deconstruct the significant interaction, a Tukey’s honestly significant difference (HSD) post-hoc test was conducted. The results of the pairwise comparisons are illustrated in Fig. 1 (*p* < .05).

A comparison among sites based on the number of MFs removed by tape tearing (Fig. 1) showed that the highest number of MFs was collected at the industrial site, followed by the urban sites 1 and 2 and the three green sites. Significant differences between sites, even of the same typology (*i.e.,* urban or green), were more pronounced in summer than in winter, where a more homogeneous MFs accumulation occurred. At each site, MFs accumulation was significantly higher in summer than in winter, apart from the green sites 1 and urban 3.

As for the length of MFs, it ranged between 50 µm to 12 mm over all sites, with the highest length found in leaf samples from industrial site. For the analysis, MFs having a length >5 mm were not considered. A comparison between the length of MFs found on the leaves collected at the two seasons (Table 2) showed that those observed in summer were significantly longer than MFs found on winter leaves.

**Table 2:** MFs length at each site and season.

**Site →**	**Industrial**	**Urban1**	**Urban2**	**Urban3**	**Green1**	**Green2**
**Season ↓**	**Number of MFs**					
	**Length (Mean ± SD)**					
**Summer**	160	92	125	84	48	54
	0.64 ± 1.3^*^	0.76 ± 0.58^*^	0.99 ± 1.10^*^	0.93 ± 1.07^*^	0.93 ± 0.94	0.7 ± 0.72
**Winter**	71	47	30	60	53	24
	0.35 ± 0.39	0.56 ± 0.43	0.43 ± 0.29	0.58 ± 0.72	0.62 ± 0.55	0.5 ± 0.22
^*^Mann Whitney’s U test (*p* < 0.05)	*p = 0.02*	*p = 0.02*	*p = 0.002*	*p = 0.001*	*p*= 0.06	*p*= 0.75

**Notes.**

Comparison according to Mann–Whitney U test (*p* < 0.05). Asterisks on the median values indicate significantly longer fibers.

### Accumulation of MFs in transplants

**Figure 2:**
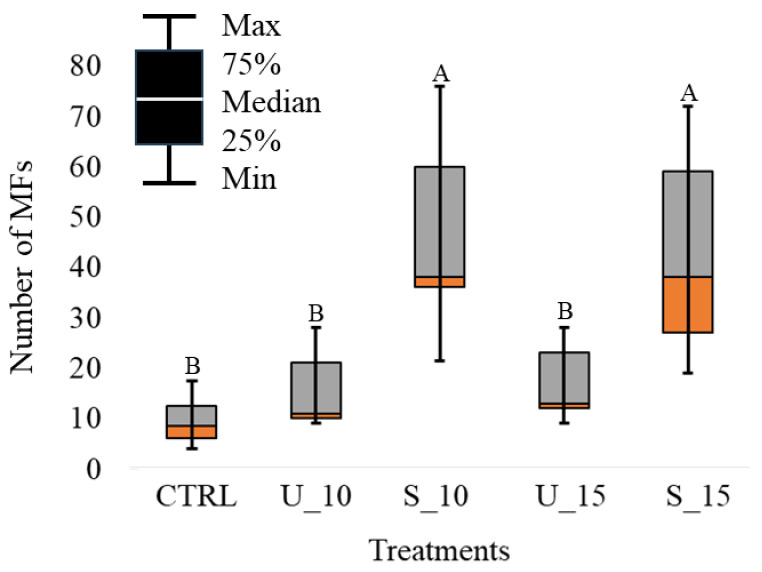
Box plots of MFs accumulated in transplants. Number of MFs in control (CTRL), sheltered (S) and un-sheltered (U) leaves counted after rainfalls. Different letters indicate significant differences according to Mann Whitney’s U test (*p* < 0.05).

Water washing of the transplants carried out before exposure significantly decreased the number of MFs; we found indeed, 20.44 ± 2.07 number of MFs in pre-washing samples *vs.* 9.11 ± 1.05 in post-washing ones (*t*-test, *p* < 0.05). The accumulation of MFs in transplanted *P. tobira* were significantly different between plants unsheltered and sheltered to the rainfall (Fig. 2); the exposure to heavy rain determined, in fact, a significant loss of MFs. Ten days exposure resulted in an adequate exposure time to ensure a clear accumulation on the leaves of *P. tobira*; no significant difference was found depending on the different exposure times (*i.e.,* 10 and 15 days) before a heavy rain event (Fig. 2).

### SEM observations

**Figure 3:**
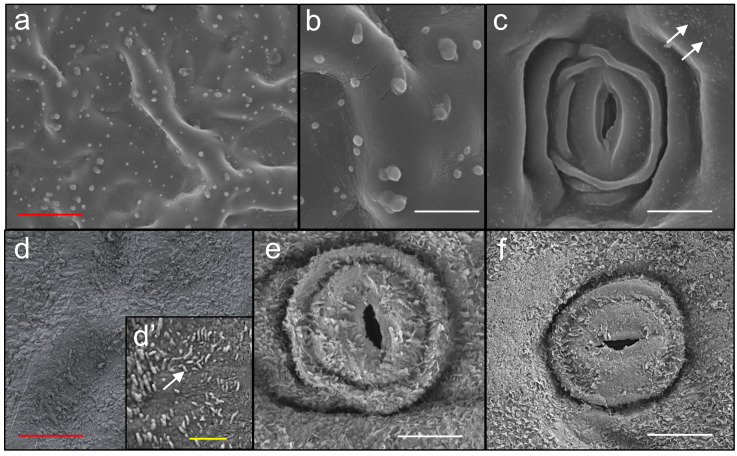
SEM observations of the leaves. Summer (A, B, C) and winter (D, E, F) leaves observed by SEM. Red, white and yellow bars are 50, 10 and 1 µm in length. White arrows indicate epicuticular waxes.

The comparison between summer and winter leaves (Fig. 3) highlighted that the first ones (a, b, and c) showed numerous glandular hairs on the upper surface, which is also characterized by irregular ridges, and sunken stomata on the lower surface, showing residual, very small epicuticular waxes (white arrows). Winter leaves instead, did not show glandular hairs, but abundant epicuticular waxes, rod-shaped on the upper surface (d, d’), and in the form of tiny scales on the lower surface (e, f). Stick-shaped epicuticular waxes were found on the upper surface of the winter leaves, and had an irregular distribution, with an average number ± SD of 16 ± 2 µm^−2^ in areas densely covered, and 2.5 ± 1.8 µm^−2^ in areas with reduced coverage (see Fig. S1). Waxy structures were 0.05 to 0.5 µm in length. Glandular hairs observed on the upper surface of summer leaves were 20.6 ± 2.2*50 µm^−2^ in number (average ± SD) and 1 to 5 µm in length.

## Discussion

In the present work we demonstrated that the leaves of *Pittosporum tobira* can be a useful biomonitor of airborne MFs in urban environment, being capable of distinguishing among the different land uses, with the highest accumulation in the industrial site and lowest in urban parks. In a previous work (Capozzi et al., 2024) we set-up a four-steps sequential extraction technique and found that the largest fraction of MFs, about 75%, was extracted by the first step, consisting in a tape tearing; therefore, we suggested this single step as a fast, and reproducible method to quantify the level of airborne MFs. In the present work we highlighted that the extraction procedure is reliable in both dry and wet seasons, despite the different morphological traits of the leaves. Notably, this single step extraction protocol could distinguish different levels of anthropogenic impact due to airborne MFs, even in sites within few kilometers from each other.

Our results only partly agree with the previous literature on plant biomonitoring. Leonard et al. (2023) did not find significant differences in MPs accumulation depending on the land use; however, they considered some plants species having different ability to intercept and retain MPs, without comparing the same species at the different sites, which could provide unclear results, since each species shows peculiar pollutants uptake and retention capacity due to different morphological traits (Capozzi et al., 2020b; Corada et al., 2021; Prigioniero et al., 2023). The same authors (Leonard et al., 2023) hypothesized a limited impact of the land use on MPs accumulation due to long-distance transport of these pollutants. Although long-range transport could have a homogenizing effect on the accumulation of MPs masking the differences due to the land uses, the results of the present study highlight that other factors can also influence the retention of these pollutants by acting on both the accumulation and the release capacity of the biomonitor. Specifically, our results suggest that the continuous supply of MFs from sources close to the monitored sites, as for industrial and urban sites, can enhance accumulation in the biomonitor, counteracting the homogenizing effect of long-distance transport. The longest MFs were found in the industrial site, which supports this hypothesis; in fact, the proximity to pollution sources, would ensure a high, continuous contribution in terms of number of longer MFs, without a parallel increase in their degradation.

In agreement with our results, Jafarova et al. (2023), using *Robinia pseudoacacia* leaflets as biomonitors of airborne MPs, found an interesting relation between accumulation and land use. In fact, a high number of microfibers, but a limited number of tire wear particles were found in urban parks, whereas an opposite trend was observed along roadside. Also including cryptogams for literature comparison, the same authors found that lichen bags exposed in Milan accumulated a higher number of MFs in the city center and periphery in comparison to city parks outside the city center (Jafarova et al., 2022). Moreover, Loppi et al. (2021) found a MPs accumulation gradient in the native lichen *Flavoparmelia caperata* sampled at different distances from a landfill damping, with the highest accumulation near to the landfill and the lowest at 1,500 m distance.

The higher accumulation of MPs during the dry season (Enyoh et al., 2019; Qian, Chong Guo & Yong Ming, 2017; Zhao et al., 2015), as well as the lower accumulation in the wet season (Zhang et al., 2020) are in line with the previous literature reports (Jahandari, 2023). Moreover, since the length of MFs accumulated in summer was significantly longer than in winter, it is likely that rainfall can induce the loss of the longest MFs. Anyhow, heavy rainfall decreases the level of MPs accumulation, as here specifically highlighted by *P. tobira* transplants. Transplant experiment also evidenced that a vigorous wash before exposure is recommended to lower the number of MFs, increasing transplant sensitivity; further, a couple of weeks is an exposure time long enough to ensure an accumulation of MPs significantly higher than pre-exposure content.

In contrast with the general trend, the seasonal variation in the accumulation and retention of MFs was not observed in two sites, Urban 3 and Green 1; this result could depend on the structure of the vegetation in the two sites, in which *P. tobira* plants used for leaf sampling were protected by the tree canopies, thus avoiding the MFs washout observed in the wet season in the other sites. In agreement with this result, it is known that the canopy of plants in urban environments can also exert a filter effect against contaminants dispersed in the atmosphere, improving air quality of the cities (McPherson et al., 2011); this has been demonstrated for several pollutants and plant species (Beckett, Freer-Smith & Taylor, 2000; De Souza et al., 2022; Oyana et al., 2017; Wannaz et al., 2013). More generally, the lack of seasonal variability in MFs accumulation at the Urban 3 and Green 1 sites suggests that the geomorphology of the territory, as well as the presence of densely wooded areas, could play a relevant role in the MFs interception and retention ability by *P. tobira*, affecting the balance between their accumulation and release.

Seasonal differences in the accumulation of MFs found in the other sites could partly depend on the different traits observed in the mature leaves in the two seasons; in fact, in the wet season leaves are covered with epicuticular waxes scale-shaped on the lower surface and stick-shaped on the upper surface. In summer instead, leaves develop on the upper surface numerous glandular hairs, which could play a role in facilitating MFs adhesion. Furthermore, despite the presence of glandular hairs only on the upper surface of the leaf, the exudates of these glands could play a role in the adhesion of the MFs on both surfaces, given the shoot architecture, with the leaves of the different layers in close contact with each other.

Other authors investigated MPs accumulation ability of different plant species (Liu et al., 2020; Perera et al., 2024) and found a noticeable variation in accumulation performance, both within and between species, suggesting that all plants can intercept and retain these pollutants, regardless leaf traits. Our work, instead, evidenced in a single species sampled at the same sites, a different ability of MFs accumulation depending on the different leaf traits observed in summer and winter, indicating that species (and its leaf traits), as well as the season, are important factors to ensure an appropriate sensitivity of the method and reliable results.

All these considerations suggest the need for further work, to precisely focus on the role of the leaves in the retention of MFs and the mechanisms affecting their uptake, retention, and release into the atmosphere. This knowledge is essential to set up a reliable, standardized methodology, which can be applied in different environmental conditions.

## Conclusions

In this work we tested the ability of *Pittosporum tobira* leaves to highlight the level of airborne MFs pollution in six sites with different land uses (industrial, urban, and green), all embedded in the urban context of Naples city. *P. tobira* leaf samples subjected to a fast, green protocol for MFs extraction, simply consisting in a tape tearing, accumulated a higher number of MFs in the industrial site, followed by urban and green sites, indicating that this species is a suitable biomonitor of airborne MFs. The accumulation of MFs was higher in summer than in winter, likely due to the water-washing effect caused by rainfall occurring in winter, and to the specific leaf traits observed in the different seasons. For the first time, *P. tobira* transplants were used to estimate the effect of pouring rain on MFs accumulation. This experiment highlighted that heavy rainfall determined a loss of MFs, specifically the longest ones, indicating that biomonitoring of airborne MFs in the wet season could lead to an underestimation of these air pollutants, also decreasing the sensitivity of the method; therefore, the comparison between biomonitoring surveys should be done considering the season. Although further studies are needed to reach a deep knowledge of plants as biomonitors of airborne MFs and set up a standardized methodology, the simple extraction method proposed in the present work could provide a feasible tool for a project of citizen science to increase people awareness of air pollution due to plastic waste.

## Supplemental Information

10.7717/peerj.20558/supp-1 Supplemental Information 1 Raw data

10.7717/peerj.20558/supp-2 Supplemental Information 2 Site coordinates

10.7717/peerj.20558/supp-3 Supplemental Information 3 Rainfall regime and wind speed during the exposure of the transplants of *Pittosporum tobira*

10.7717/peerj.20558/supp-4 Supplemental Information 4 SEM observations of the leaves Upper surface of the winter leaves (a) covered with numerous stick-shaped waxy structures. Upper surface of summer leaves (b) covered with glandular hairs.

## Additional Information and Declarations

### Data Availability

The following information was supplied regarding data availability: The raw measurements are available in the Supplemental Files.
