# Supplementary material for: A green, fast protocol to estimate the accumulation of airborne anthropogenic microfibers in Pittosporum tobira in urban areas: effects of season and rainfall
Source: PeerJ. 2026 Jan 14;14:e20558. doi: 10.7717/peerj.20558 (PMC12811961; doi:10.7717/peerj.20558)
Supplement: Supplemental Information 2 [file peerj-14-20558-s002.docx]

**Table S1.** Site coordinates.

| **SITE CODE** | **UTM - 33T** | |
| --- | --- | --- |
|  | **E** | **N** |
| Industrial | 441581.533 | 4522366.495 |
| Urban_1 | 442357.312 | 4521633.697 |
| Urban_2 | 434947.137 | 4522206.89 |
| Urban_3 | 434247.247 | 4524864.304 |
| Green_1 | 436904.991 | 4524866.877 |
| Green_2 | 430829.289 | 4516656.277 |
