# Supplementary material for: A green, fast protocol to estimate the accumulation of airborne anthropogenic microfibers in Pittosporum tobira in urban areas: effects of season and rainfall
Source: PeerJ. 2026 Jan 14;14:e20558. doi: 10.7717/peerj.20558 (PMC12811961; doi:10.7717/peerj.20558)
Supplement: Supplemental Information 3 [file peerj-14-20558-s003.docx]

**Table S2.** Rainfall regime and wind speed during the exposure of the transplants of *Pittosporum tobira*.

| **Day** | **January 2025** | | **February 2025** | |
| --- | --- | --- | --- | --- |
|  | **R*** | **W** | **R** | **W** |
| 1 | **0** | **1** | **0** | **1** |
| 2 | **0** | **1** | **0** | **3** |
| 3 | **5.8** | **3** | **0** | **2** |
| 4 | **0.8** | **2** | **0** | **2** |
| 5 | **0** | **2** | **0** | **2** |
| 6 | **0** | **3** | **0** | **1** |
| 7 | **0** | **3** | **0** | **2** |
| 8 | **0** | **1** | **2** | **2** |
| 9 | **0.6** | **4** | **0** | **2** |
| 10 | **0** | **2** | **0** | **2** |
| 11 | **34.8** | **2** | **0** | **1** |
| 12 | **8.8** | **6** | **0** | **1** |
| 13 | **0** | **5** | **0.4** | **1** |
| 14 | **0** | **5** | **6** | **3** |
| 15 | **0** | **3** | **15.6** | **2** |
| 16 | **0.2** | **3** | **0** | **2** |
| 17 | **0** | **5** | **0** | **1** |
| 18 | **2.4** | **5** | **0** | **2** |
| 19 | **0** | **3** | **0** | **2** |
| 20 | **7.2** | **2** | **0** | **2** |
| 21 | **8.6** | **1** | **0** | **2** |
| 22 | **0.2** | **1** | **0** | **1** |
| 23 | **0** | **1** | **0** | **1** |
| 24 | **0** | **1** | **1.8** | **1** |
| 25 | **0** | **1** | **4** | **3** |
| 26 | **0** | **2** | **34.8** | **2** |
| 27 | **0** | **2** | **0.2** | **1** |
| 28 | **2.8** | **4** | **0** | **2** |
| 29 | **6** | **2** |  |  |
| 30 | **0** | **1** |  |  |
| 31 | **0** | **1** |  |  |

*R= rainfall (mm)

W= wind speed according to Beaufort scale (0 to 12)

Start of exposure

End of exposure
