# Supplementary figures and images for: A green, fast protocol to estimate the accumulation of airborne anthropogenic microfibers in Pittosporum tobira in urban areas: effects of season and rainfall
Source: PeerJ. 2026 Jan 14;14:e20558. doi: 10.7717/peerj.20558 (PMC12811961; doi:10.7717/peerj.20558)

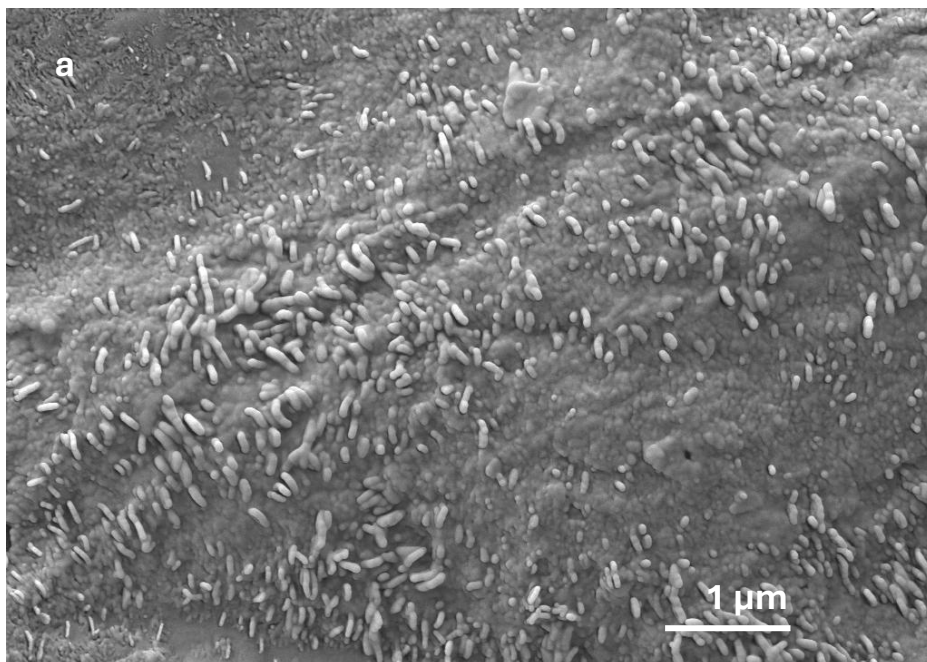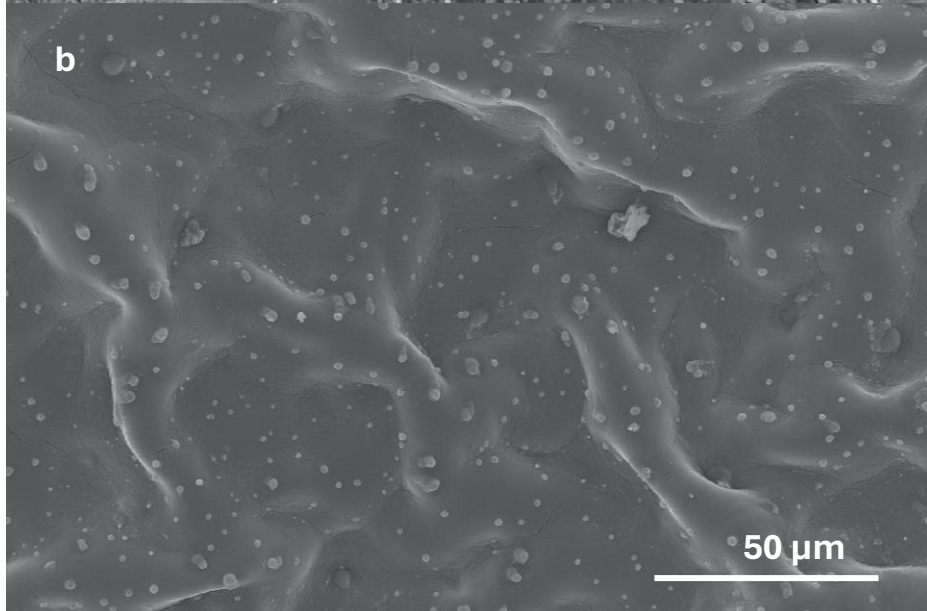

Supplement: Supplemental Information 4 — Upper surface of the winter leaves (a) covered with numerous stick-shaped waxy structures. Upper surface of summer leaves (b) covered with glandular hairs. [file peerj-14-20558-s004.pdf]
